# To Study the Impact of Tooth Sectioning on Postoperative Pain, Swelling and Trismus After Surgical Extraction of Impacted Mandibular Third Molars

**DOI:** 10.7759/cureus.51207

**Published:** 2023-12-28

**Authors:** Tanveer Karpe, Arshiya Sanober, Fazil A Nasyam, Sureddy Soumya, Swetcha Seethamsetty, Godvine Sarepally

**Affiliations:** 1 Department of Oral and Maxillofacial Surgery and Diagnostic Science, Faculty of Dentistry, Taif University, Taif, SAU; 2 Department of Oral and Maxillofacial Surgery, Government Dental College and Hospital, Hyderabad, IND; 3 Department of Oral and Maxillofacial Surgery and Diagnostics Sciences, College of Dentistry, Prince Sattam Bin Abdul Aziz University, Al Karj, SAU; 4 Department of Oral and Maxillofacial Surgery, Swetha Reddy Multi Speciality Dental Clinic, Hyderabad, IND; 5 Department of Oral and Maxillofacial Surgery, Leela Dental Specialities, Kakinada, IND; 6 Department of Oral and Maxillofacial Surgery, Panineeya Mahavidyalaya Institute of Dental Sciences, Hyderabad, IND

**Keywords:** third molars, surgical extraction, trismus, swelling, postoperative pain

## Abstract

Aim: To study the impact of tooth sectioning on postoperative pain, swelling, and trismus after surgical extraction of impacted mandibular third molars.

Materials and methods: The present research was conducted on a sample of 100 individuals who were in good health. The participants had an average age of 28 years and were seeking treatment at the Department of Oral and Maxillofacial Surgery for the extraction of impacted mandibular third molars. The participants were allocated randomly to one of the two experimental conditions. The patients in Group A are receiving a surgical procedure to remove the mandibular third molar without the need for tooth sectioning. The study focuses on patients classified as Group B who are having a surgical procedure for the extraction of the mandibular third molar using dental sectioning.

Results: The Group B patients saw a notable decrease in pain intensity on the third and seventh days after the surgery. The mean difference in pain scores was 4.15±0.54 and 1.69±0.11, respectively, indicating statistically significant findings with a p-value of less than 0.05. The study observed statistically significant differences in swelling between the 1st, 3rd, and 7th postoperative days for group II. The mean differences were recorded as 149.85±5.86, 119.25±4.22, and 107.52±, respectively. The significance level was determined to be P<0.05. The study observed that the degree of mouth opening in Group B exhibited a significant rise on the 3rd and 7th postoperative days, with a mean difference of 40.87±3.69 and 43.15±3.29, respectively, as compared to Group A. This difference was found to be statistically significant, with a p-value of less than 0.05.

Conclusion: The findings of our research indicate that the technique of sectioning the tooth is the preferable approach for surgical extraction of impacted third molars, as it effectively reduces the occurrence of post-operative problems.

## Introduction

An impacted tooth refers to a tooth that is unable to emerge into the dental arch within the expected timeframe due to factors such as misalignment, insufficient space, or an obstruction along its route of eruption. The extraction of impacted mandibular third molars is a frequently carried out dental alveolar technique, and it is accompanied by a range of postoperative complications [1]. The postoperative phase after the surgical extraction of the third molar (wisdom teeth) typically results in discomfort (pain), trismus, and edema. The aetiology of these disorders is multifactorial and intricate, stemming from an inflammatory cascade triggered by surgical trauma [2].

According to studies conducted, individuals who suffer pain, edema, and trismus after wisdom tooth surgery have a threefold larger impact on their quality of life compared to people who do not exhibit these symptoms [3]. Therefore the surgeon should understand the need of the hour and enhanced management of pain, edema, and trismus in patients undergoing third molar surgery. Limited research is conducted on the patients' expectations about the outcome of such surgical procedures, despite the existence of studies documenting patients' impressions of healing after third molar surgery [4].

The occurrence of postoperative pain, swelling, and trismus subsequent to third molar surgery is believed to be a result of the inflammatory response that directly and indirectly promptly follows the surgical procedure [5]. These symptoms definitely have a detrimental impact on the patient's quality of life both physically, emotionally, and psychologically after the surgery [6]. Therefore, it is crucial to assess the complexity of the surgical process and communicate with the patient about the resulting potential problems. The relationship between surgical difficulty and postoperative complications has been documented in various studies [7]. However, some researchers argue that relying solely on radiologic methods to assess difficulty may not accurately estimate the actual level of difficulty, and that intraoperative estimation is necessary [8]. Several studies have included operating time and surgical skill as factors to assess the level of difficulty [5-8]. The existing body of research provides more insights into the assessment of the complexity of third molar surgery. However, the conclusions drawn from these studies are inconsistent, and there exists a significant degree of variability in the components that contribute to this assessment [8].

A comprehensive literature review was conducted to identify key variables consistently identified as factors influencing surgical difficulty, as measured by operating time [9]. This review provides the most up-to-date evidence relevant to clinical practise regarding the assessment and surgical treatment of impacted mandibular third molars. Age, surgical method, number of teeth extracted, depth angle, and root shape have been identified as the most consistent factors of difficulties in previous studies [9]. Numerous methods have been developed with the aim of facilitating the effective extraction of the third molar while minimising associated difficulties [9,10]. The selection of surgical approach varies across surgeons and has been associated with the occurrence of nerve injury, as well as the intensity of postoperative discomfort and edema [11]. The objective of our research is to evaluate the quality of life experienced by patients who have undergone surgical extraction of impacted mandibular third molars, specifically comparing the outcomes of tooth sectioning to full odontectomy. Hence, it is essential to exert all possible endeavours in order to minimise the occurrence of postoperative complications and enhance overall patient satisfaction along with improving patient quality of life associated.

## Materials and methods

The present research was conducted on a sample of 100 individuals who were in good health. The participants had an average age of 28 years and were seeking treatment at the Department of Oral and Maxillofacial Surgery, S.B. Patil Dental College and Hospital, Bidar, Karnataka, India. Individuals undergoing the extraction of impacted mandibular third molars were designated as patients. The study was carried out from March to July 2015 and the research protocol underwent a thorough assessment and received approval from the Institutional Review Board at S.B. Patil Dental College and Hospital (SBPDCH/2015/200).

The study goal was communicated to all patients, and their agreement was acquired to facilitate smooth completion and comprehension. Before the commencement of the study, patient informed consent was obtained. The participants were allocated randomly to one of the two experimental conditions. The patients in Group A underwent a surgical procedure to remove the mandibular third molar without the need for tooth sectioning. The study focuses on patients classified as Group B who are having a surgical procedure for the extraction of the mandibular third molar using dental sectioning.

Inclusion criteria

The study focuses on individuals classified as American Society of Anesthesiologists (ASA) I who are scheduled to receive surgical extraction of Mesioangular impacted mandibular third molars. The categorization system developed by Pell and Gregory, specifically focusing on Class I and II fractures, Position A, is of academic interest.

Exclusion criteria

The study population consisted of individuals presenting under ASA II, III, and IV with impacted Mesioangular (class III), vertical, horizontal, and Distoangular mandibular third molars. Individuals who have systemic diseases that make them more susceptible to developing local infections, such as diabetes mellitus, AIDS, or undergoing concomitant cancer treatment. Individuals who exhibit local variables that increase their susceptibility to infections, such as periapical disease, cysts, neoplastic lesions, or a history of radiation on the mandible.

Methodology

Sample selection: The patients who meet the criteria for eligibility are allocated in a random manner to either Group A, which involves the non-sectioning of the tooth, or Group B, which involves the sectioning of the tooth.

Evaluation criteria radiologically: Periapical radiographs intraorally were obtained according to Winter’s and Pell and Gregory's classification. Mesioangular impacted mandibular third molar in relation to ramus was categorized as Class I and Class II; Depth: position A-high occlusal level; Ramus relation: Class I and Class II.

The oral cavity was prepared with a solution of povidone-iodine. The administration of anaesthesia was achieved by using a solution containing 2% lignocaine hydrochloride and 1:200000 adrenaline. This was accomplished by the use of inferior alveolar nerve block, lingual nerve block, and long buccal nerve block techniques. A surgical incision known as Ward's incision, or Ward's incision with distal extension, was performed. Subsequently, the mucoperiosteal flap was carefully reflected, allowing for the exposure of the underlying bone. The assessment of intraoral periapical radiographs was conducted using Winter's and Pell and Gregory's classification systems. The impacted tooth in question was identified as a Mesioangular mandibular third molar, and its connection with the ramus was classified as either Class I or Class II. The only position that was considered in regard to depth was position A.

Group A: The extraction of bone was performed by creating grooves on the outside and back sides of the teeth using a straight fissure bur number 701 made of stainless steel, if needed. Following the appropriate amount of bone reduction, the tooth was then raised and extracted from the socket.

Group B: In this study, a conventional surgical technique was employed to remove bone from the buccal and distal regions of the impacted tooth. Furthermore, an odontectomy was performed by positioning a bur in the buccal groove and subsequently moving it in a bucco-lingual direction along the longitudinal axis of the tooth, resulting in the division of the tooth into two halves. Subsequently, the tooth was raised and extracted from its socket. In conclusion, the socket irrigation procedure included the use of povidone-iodine and saline for both Group I and Group II. The bony borders were smoothed using bone files, and the socket was irrigated to eliminate any bone debris. A state of complete hemostasis was attained prior to the closure of the wound. The incision was sutured with 3-0 silk thread, and the patient received postoperative instructions. All patients received antibiotic treatment consisting of oral amoxicillin 500 mg given every eight hours, as well as oral metronidazole 400 mg.

**Figure 1:**
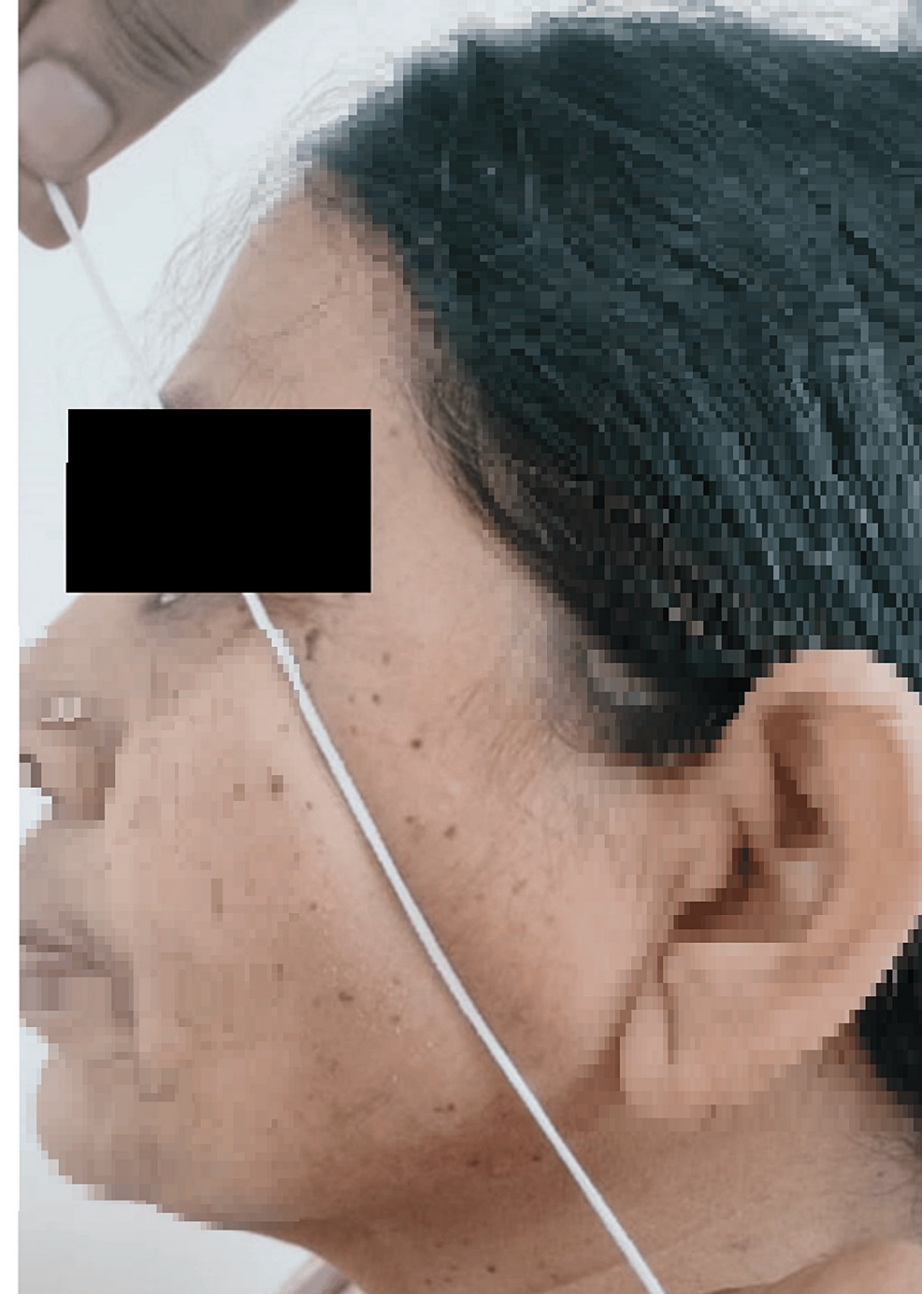
S1-distance from the outer canthus of the eye to the angle of the mandible

**Figure 2:**
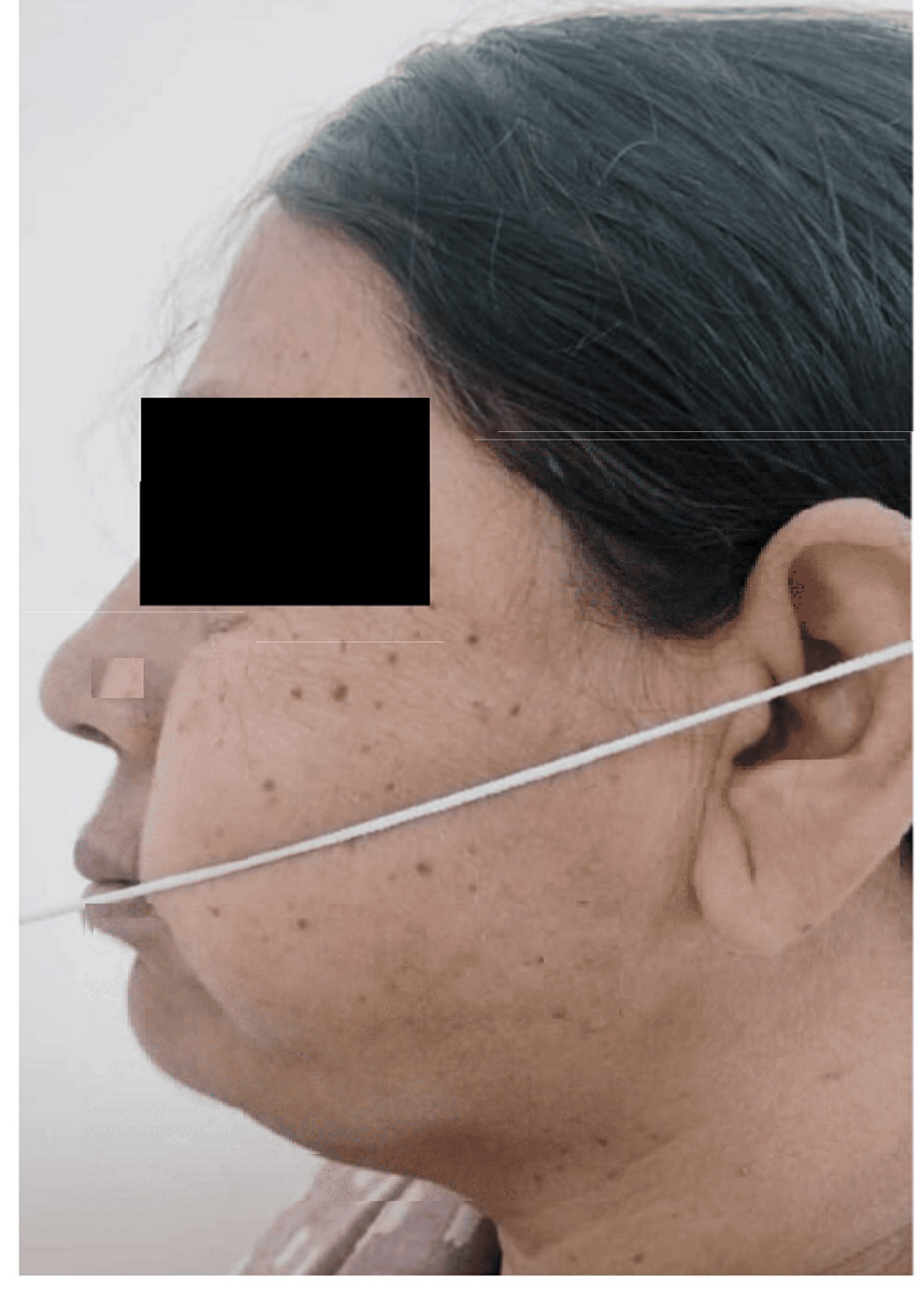
S2-distance from the tragus of the ear to the corner of the mouth

**Figure 3:**
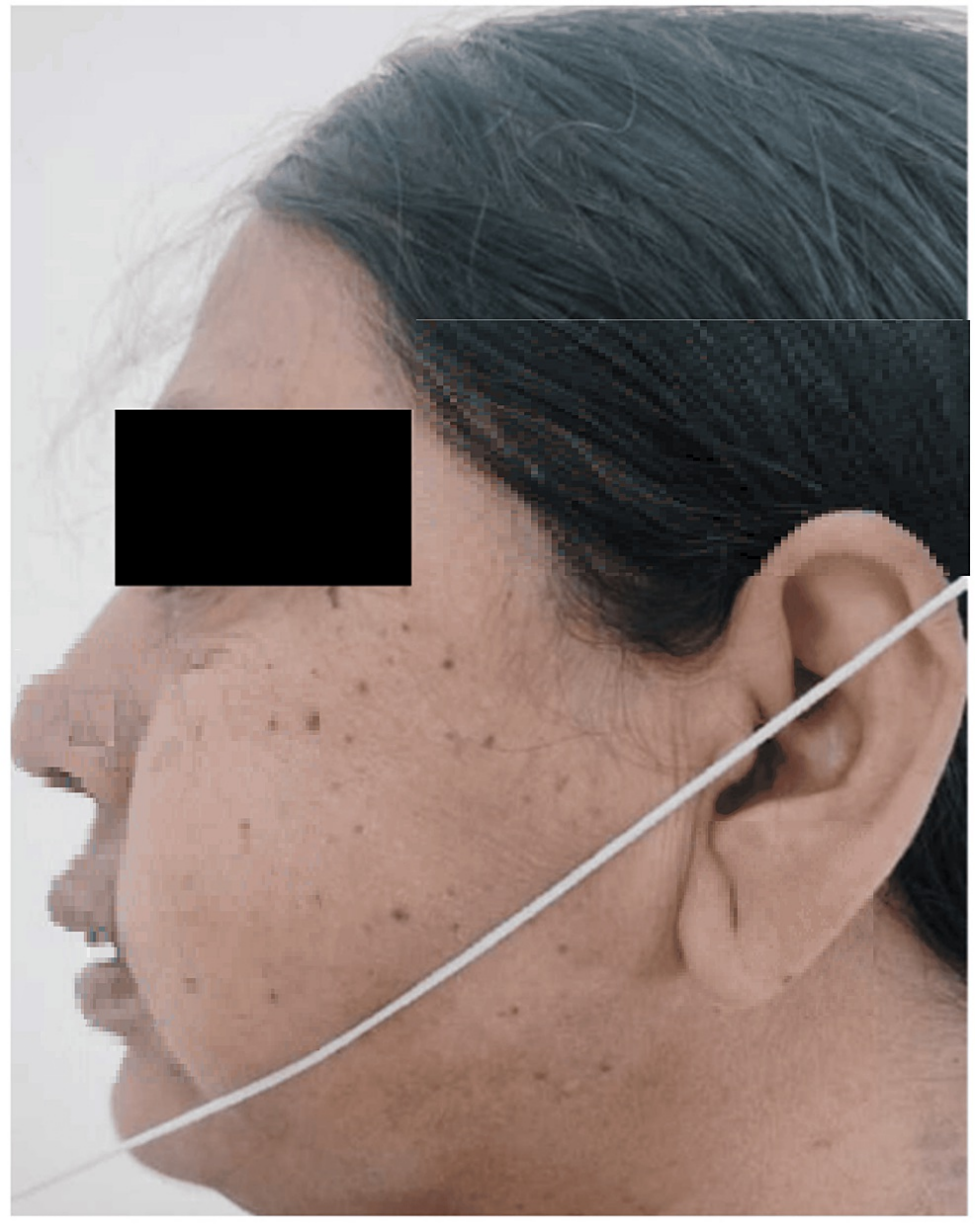
S3-distance from the tragus of the ear to the soft tissue pogonion

**Figure 4:**
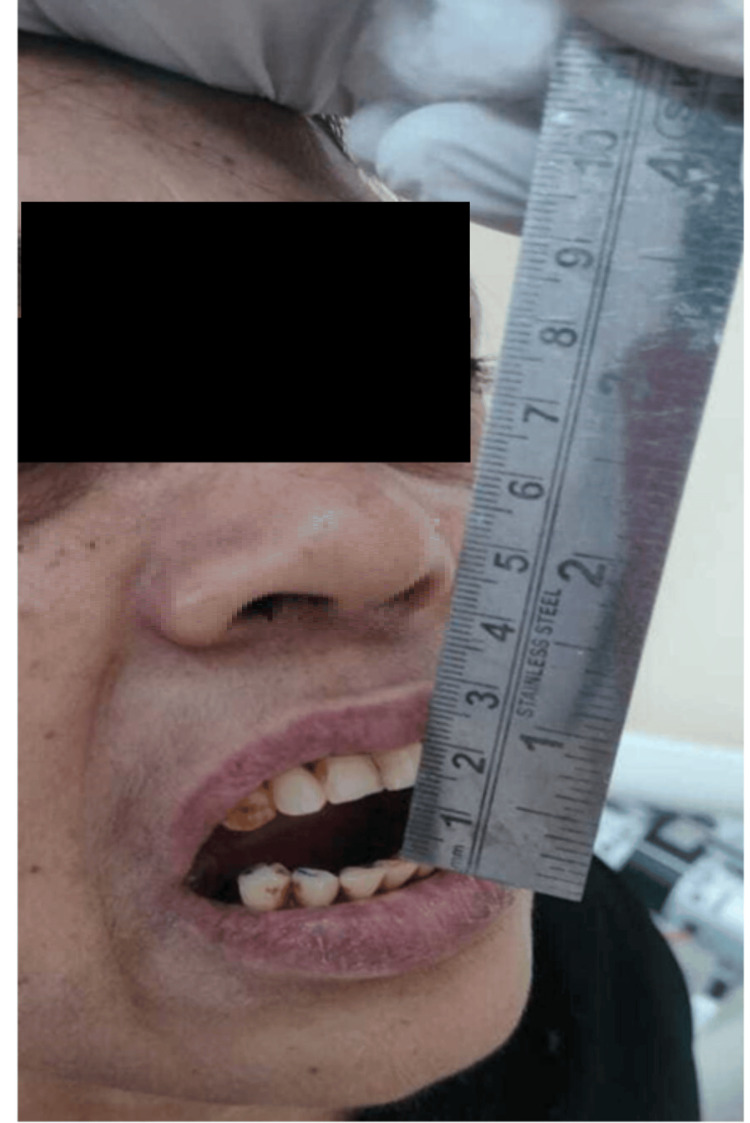
S4-distance in millimetres between the incisal margins of the upper and lower central incisors

Additionally, patients were provided with one mole of pain relief medication every eight hours. The patients had evaluation by a single independent observer in the postoperative period, namely on the first, third, and seventh days. The observer assessed parameters related to discomfort, edema, and trismus. Pain assessment was conducted via a visual analogue scale (VAS) measuring 10 cm in length. The VAS used a scoring system ranging from 0 to 10, where 0 represented the absence of pain, 5 indicated a state of moderate discomfort, and 10 denoted the most severe level of pain imaginable. The craniometric approach was used to assess facial swelling, which included measuring the distance from the outer canthus of the eye to the angle of the mandible (S1) (Figure 1), the distance from the tragus of the ear to the corner of the mouth (S2) (Figure 2), and the distance from the tragus of the ear to the soft tissue pogonion (S3) (Figure 3). The measurements were acquired using a string and were recorded in mm on a conventional graded scale. The sum of S1, S2, and S3 was calculated and referred to as the variable S, representing the amount of swelling. The measurement of maximum mouth opening was conducted by determining the distance in mm between the incisal margins of the upper and lower central incisors (Figure 4).

## Results

**Figure 5:**
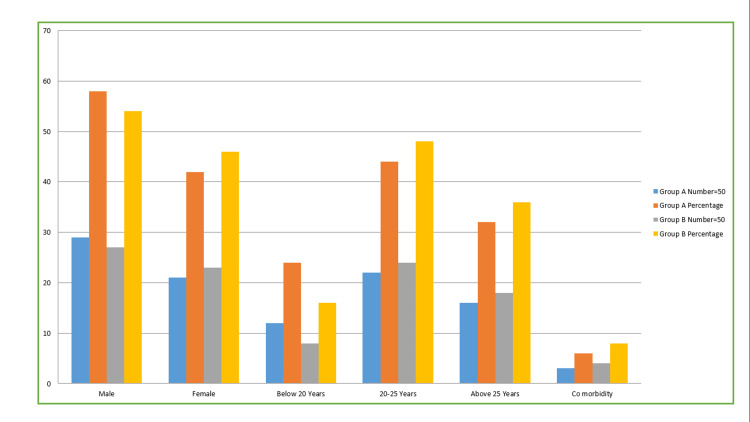
Basic profile of the patients Group A consisted of 29 men (58%) and 21 women (42%), while Group B consisted of 27 men (54%) and 23 women (46%). With respect to the age of patients, in Group A, 12(24%) were below 20 years, 22 (44%) were between 20-25 years, 16 (32%) were above 16 years, and three (6%) were with co-morbidity. In Group B, eight (16%) were below 20 years, 24 (48%) were between 20-25 years, 18 (36%) were above 16 years, and four (8%) were with co-morbidity.

The current research included a sample size of 100 patients who required surgical extraction of impacted mandibular third molars. Among these patients, the basic profile of the patients is distributed as follows: Group A consisted of 29 men (58%) and 21 women (42%), while Group B consisted of 27 men (54%) and 23 women (46%). With respect to the age of patients, in Group A, 12 (24%) were below 20 years, 22 (44%) were between 20-25 years, 16 (32%) were above 16 years, and three (6%) were with co-morbidity. In Group B, eight (16%) were below 20 years, 24 (48%) were between 20-25 years, 18 (36%) were above 16 years, and four (8%) were with co-morbidity (Table 1, Figure 5).

**Table 1:** Basic profile of the patients Data is represented in Percentage. The table presents the basic profile of the patients. Group A consisted of 29 men (58%) and 21 women (42%), while Group B consisted of 27 men (54%) and 23 women (46%). With respect to the age of patients, in Group A: 12 (24%) were below 20 years, 22 (44%) were between 20-25 years, 16 (32%) were above 16 years, and three (6%) were with co-morbidity. In Group B, eight (16%) were below 20 years, 24 (48%) were between 20-25 years, 18 (36%) were above 16 years, and four (8%) were with co-morbidity.

	Group A		Group B		P value
	Number=50	Percentage	Number=50	Percentage	0.21
Gender					
Male	29	58	27	54	
Female	21	42	23	46	
Age (Years)					0.15
Below 20	12	24	8	16	
20-25	22	44	24	48	
Above 25	16	32	18	36	
Co-morbidity	3	6	4	8	

The pain, swelling, and mouth opening ratings for both groups on the first, third, and seventh postoperative days are respectively demonstrated in Tables 1-4. The data underwent statistical analysis using the ANOVA test in SPSS software, version 25.0 (IBM Corp., Armonk, NY).

**Table 2:** ANOVA test of significance for pain for Group A and B Data is represented in Mean±SD. The table provides a comparative analysis of pain levels between Group A and Group B. The Group B patients experienced a notable decrease in pain intensity on the third and seventh days after the surgery. The mean difference in pain scores was 4.15±0.54 and 1.69±0.11, respectively, indicating statistically significant findings with a p-value of less than 0.05.

Pain score	Group A		Group B		P value
Day	Mean	Sd	Mean	Sd	
1^st^	6.85	0.88	6.11	0.77	0.08
3^rd^	4.87	0.63	4.15	0.54	0.03
7^th^	2.15	0.27	1.69	0.11	0.02

**Table 3:** ANOVA test of significance for swelling Data is represented in Mean±SD. This table provides the results of the comparative analysis of swelling between Group A and Group B. The study observed statistically significant differences in swelling between the 1st, 3rd, and 7th postoperative days for group II. The mean differences were recorded as 149.85±5.86, 119.25±4.22, and 107.52±, respectively. The significance level was determined to be P<0.05.

Swelling	Group A		Group B		P value
Days	Mean	Sd	Mean	Sd	
1^st^	157.52	6.35	149.85	5.86	0.03
3^rd^	126.98	5.85	119.25	4.22	0.01
7^th^	111.15	4.69	107.52	3.85	0.01

**Table 4:** ANOVA test of significance for mouth-opening Data is represented in Mean±SD. This table provides the analysis of mouth-opening measurements between Group A and Group B. The study observed that the degree of mouth opening in group B exhibited a significant rise on the third and seventh postoperative days, with a mean difference of 40.87±3.69 and 43.15±3.29, respectively, as compared to Group A. This difference was found to be statistically significant, with a p-value of less than 0.05.

Mouth opening	Group A		Group B		P value
Day	Mean	Sd	Mean	Sd	
1^st^	36.85	3.63	37.85	4.63	0.06
3^rd^	39.52	3.44	40.87	3.69	0.02
7^th^	42.11	3.47	43.15	3.29	0.02

**Figure 6:**
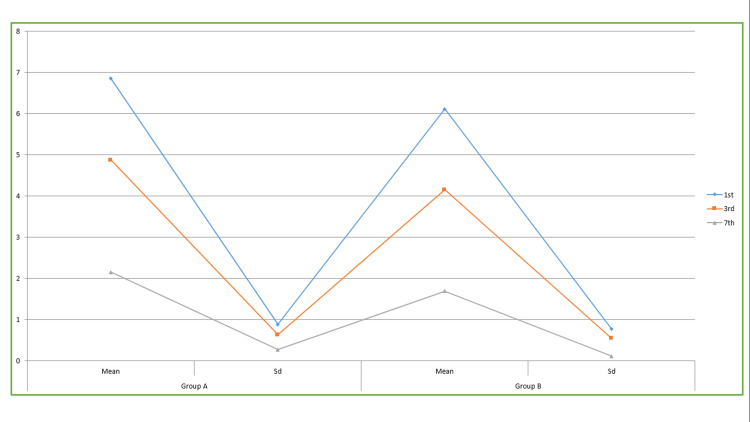
Pain score in patients This figure provides a comparative analysis of pain levels between Group A and Group B. Group B patients experienced a notable decrease in pain intensity on the third and seventh days after the surgery. The mean difference in pain scores was 4.15±0.54 and 1.69±0.11, respectively, indicating statistically significant findings with a p-value of less than 0.05.

Table 2 and Figure 6 provide a comparative analysis of pain levels between Group A and Group B. The Group B patients experienced a notable decrease in pain intensity on the third and seventh days after the surgery. The mean difference in pain scores was 4.15±0.54 and 1.69±0.11, respectively, indicating statistically significant findings with a p-value of less than 0.05. 

**Figure 7:**
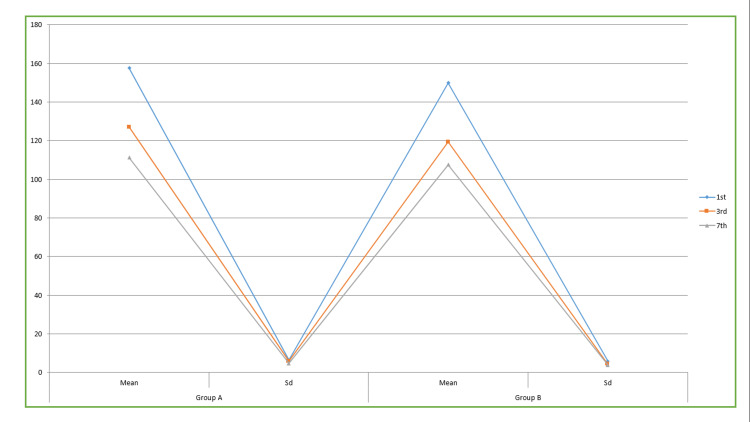
Score for swelling of the patients This figure provides the results of the comparative analysis of swelling between Group A and Group B. The study observed statistically significant differences in swelling between the first, third, and seventh postoperative days for Group B. The mean differences were recorded as 149.85±5.86, 119.25±4.22, and 107.52±, respectively. The significance level was determined to be p<0.05.

Table 3 and Figure 7 provide the results of the comparative analysis of swelling between Group A and Group B. The study observed statistically significant differences in swelling between the first, third, and seventh post-operative days for Group B. The mean differences were recorded as 149.85±5.86, 119.25±4.22, and 107.52±, respectively. The significance level was determined to be p<0.05.

**Figure 8:**
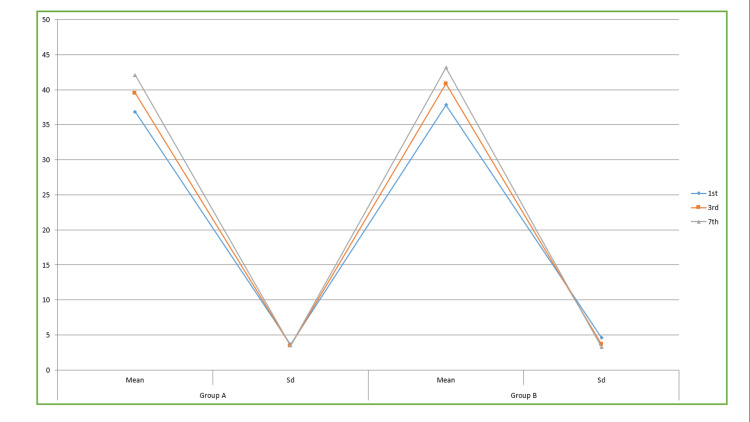
Mouth opening of the patients The figure provides the analysis of mouth-opening measurements between Group A and Group B. The study observed that the degree of mouth opening in Group B exhibited a significant rise on the third and seventh postoperative days, with a mean difference of 40.87±3.69 and 43.15±3.29, respectively, as compared to Group A. This difference was found to be statistically significant, with a p-value of less than 0.05.

Table 4 and Figure 8 provide the analysis of mouth-opening measurements between Group A and Group B. The study observed that the degree of mouth opening in Group B exhibited a significant rise on the third and seventh postoperative days, with a mean difference of 40.87±3.69 and 43.15±3.29, respectively, as compared to Group A. This difference was found to be statistically significant, with a p-value of less than 0.05.

## Discussion

Extraction of impacted mandibular third molars leads to a postoperative inflammatory response often marked with discomfort, swelling, and different degrees of limited mouth opening. The duration of surgical recovery with healing takes around seven days, during which side effects like inflammation, discomfort (pain), and trismus are the major manifestations that are experienced by the patients. This study aimed to investigate the correlation between the postoperative inflammatory response and the level of difficulty associated with the surgical extraction of impacted third molars.

The objective of this research was to assess the postoperative progression of pain, edema, and mouth opening after the extraction of impacted mandibular third molars using both non-sectioning and sectioning procedures with a focus on duration and technique of surgery. The study also tries to identify the most reliable indicator of operative difficulty that correlates with postoperative inflammation. The surgical approach used for the extraction of third molars may vary across patients, contingent upon the specific form of impaction encountered [12]. The kind of impaction influences the technique of extraction required for tooth extraction during surgery, suggesting the estimated level of extraction difficulty. Glenn et al. [13] in their study introduced a tooth division approach as a means of extracting an impacted tooth, highlighting the potential benefits of minimising the surgical area. Due to the limited or negligible posterior dental procedures, the incisions required are comparatively less substantial and this phenomenon results in a reduction in postoperative edema. The process of bone removal is either stopped or significantly decreased, reducing the duration of the operation, along with limiting the degree of trismus. Trismus is mostly caused by damage to the ligaments of the temporomandibular joint and muscles of mastication as a consequence of vigorous tooth elevation in the absence of harm or injury seen in the neighbouring teeth and underlying bone structure. With respect to the jaw fractures that occur during the impaction procedures, the majority of mandibular fractures occur due to external forces causing upward displacement, often involving vertical impactions that come into contact with the second molars.

Another problem patients experience during the removal of an impacted third molar removal is lip paraesthesia. This problem of lip numbness often occurs as a consequence of substantial mechanical strain exerted on the tooth's roots, leading to compression of the mandibular nerve. The problem of lip paraesthesia can be resolved by adopting a procedure that involves gentle application of pressure to detach the roots from the nerve. The establishment of pain measurement poses challenges due to the multivariate nature of pain intensity and patient perception. According to Pedersen A [14], pain has been identified as a significant contributing factor to the reduction in mouth opening after the extraction of impacted mandibular third molars. In contrast, our research found a decrease in mouth opening on the initial post-operative day, followed by an increase in pain threshold on postoperative days 1 and 3. This phenomenon may be attributed to the acute and strong pain that patients feel in the early postoperative period. Subsequently, the mouth opening exhibited a progressive increase by postoperative day 7, ultimately reverting to its initial baseline state. The pain experienced by over 20 patients showed a progressive decrease starting from the third day after the surgical procedure. According to Milles et al. [12], participants in their research reported experiencing significant pain on the second day after surgery. In contrast, our study revealed that participants suffered severe pain on the third day post-surgery.

According to the findings of Bosch et al. [15] and Van Gool et al. [16], it was observed that the onset of pain occurred more rapidly compared to swelling, with discomfort reaching its peak within a short period of two to three days. The concurrent occurrence of discomfort and swelling ultimately resulted in the development of trismus. By the seventh day, there was a noticeable and consistent improvement in the mouth opening. Chiapasco et al. [17] highlighted the significance of excessive bone guttering resulting in the escalation of surgical discomfort. In our investigation, it was observed that the surgical extraction of the Mesioangular impacted lower third molar teeth in class II cases required a greater amount of bone removal in comparison to class I cases. Among a cohort of 100 patients, it was observed that 50 individuals presented with Class II Mesioangular impacted teeth. On postoperative days 1 and 3, the patients reported a pain VAS score of 6. According to the findings of Shevel et al. [18], it has been proposed that the use of smaller incisions leads to a decrease in post-operative discomfort.

According to Srinivas et al. [19], the occurrence of surgical edema is a common consequence after the extraction of impacted third molars. Typically, postoperative swelling tends to peak between two to three days after the procedure, and it is expected to diminish by the fourth day, with full resolution occurring by the seventh day. However, in the present investigation, it was observed that the edema reached its maximum level on postoperative day 3 (POD 3) and then decreased by postoperative day 7 (POD 7). In a research conducted by Shugars et al. [20], it was observed that the edema reached its maximum level on postoperative day 2 in 46% of the patients. In the current investigation, it was shown that 80% of the patients exhibited edema on postoperative day 3 (POD 3). The findings of Pedersen A [14] and our research indicates that there is no significant link between edema and the duration of the surgical procedure. The potential cause of this phenomenon might be attributed to the heightened manipulation of tissue or the use of buccal retraction techniques during the surgical extraction of the tooth.

White et al. [21] performed research on health-related quality of life (HRQL) to evaluate the outcomes and recovery of patients after undergoing third molar extraction. The examination of these results would likely assist the doctor in future endeavours to implement a methodical assessment and adjust the surgical approach according to the specific impaction type and preoperative status of the impacted third molar. According to Sortino et al. [22], the enlargement of the incision and manipulation of tissue has the potential to impact the magnitude of swelling and mouth opening. In the present research, all participants had either Ward's incision or Ward's incision with distal extension for the purpose of surgically extracting impacted lower third molars. However, it was observed that only 30 out of the total 100 patients experienced little limitation in mouth opening on postoperative day 7. Typically, during the process of performing a ward's incision, the distal extension that is placed when surgically extracting impacted teeth may extend laterally to either the retromolar trigone or the external oblique ridge. With this the anatomical landmarks are served at the points of attachment of insertion of temporalis muscle tendon resulting in trismus or performing extensive bone ostectomy in this particular anatomical location may also sometime result in this clinical scenario of Trismus. The presence of trismus after a surgical procedure is often linked to oral surgical treatments conducted in close vicinity of the ramus and mandibular angle. There exists a clear correlation between the degree of trismus and the severity of tissue damage caused and bone damage. The severity of trismus is heightened during the first 48 hours post-surgery, thereafter exhibiting a steady amelioration and complete cure within one week following the surgical procedure.

According to Stanley MF [23], it has been elucidated that needle piercing does not induce trismus. However, in cases where the needle tip inadvertently causes trauma to the periosteum, it becomes barbed resulting in tearing of the muscle fibres of the medial pterygoids during retrieval, subsequently inducing muscular spasm and ultimately resulting in trismus. According to White et al. [21], the act of surgery initiates a series of events in which inflammatory mediators are released, leading to a temporary constriction of arterioles followed by vasodilation, increased blood flow, heightened permeability of post-capillary venules, and the leakage of fluid into the adjacent tissue. Trismus is a condition that arises due to the contraction of these muscle fibres subsequent to an inflammatory process [24-25].

The limitation of the study revolves around orientating the surgeons in their decision-making of extraction of the impacted tooth with ease as their decision can affect the post-operative sequelae of problems for the patient. Orienting surgeons is paramount important as it can increase their comprehension about the exact influence of sectioning or non-sectioning of impacted third molar. The skills and the training received mainly influence the operator’s decision to adopt the operative procedure.

## Conclusions

Pain, swelling, and trismus are multifactorial and depend on various pre-operative factors like the angulation of the tooth, depth, space available, and position in relation to the external oblique ridge. These postoperative complications also depend on the number of needle prick attempts, incision type, mucoperiosteal flap and technique of removal of the tooth. The findings of our research indicate that the technique of sectioning the tooth is the preferable approach for surgical extraction of impacted third molars, as it effectively reduces the occurrence of post-operative problems.
